# Predictors of Seniors’ Interest in Assistive Applications on Smartphones: Evidence from a Population-Based Survey in Slovenia

**DOI:** 10.3390/ijerph16091623

**Published:** 2019-05-09

**Authors:** Andraž Petrovčič, Sebastiaan Peek, Vesna Dolničar

**Affiliations:** 1 Faculty of Social Sciences, University of Ljubljana, 1000 Ljubljana, Slovenia; vesna.dolnicar@fdv.uni-lj.si; 2 School of Social and Behavioral Sciences, Department of Tranzo, Tilburg University, 5000 LE Tilburg, The Netherlands; research@sebastiaanpeek.nl

**Keywords:** acceptance factors, assistive applications, C-TAILS model, population-based survey, seniors, smartphones

## Abstract

Assistive applications (apps) on smartphones could contribute to a better quality of life for seniors living independently at home. At present, there is a lack of empirical evidence of seniors’ acceptance of such apps. The Cycle of Technology Acquirement by Independent-Living Seniors (C-TAILS) model was recently proposed for studying the interplay between acceptance factors by integrating the personal, social and technological domains of seniors’ daily lives. This study aimed to explore how four groups of factors, clustered in accordance with the C-TAILS model, predict seniors’ interest in assistive apps, on a representative sample of the Slovenian population aged 55 years or older. The 617 respondents, who were contacted though a telephone survey, answered a questionnaire about their interest in three groups of assistive apps and four groups of potentially associated acceptance factors. Three linear regression models were used to analyse the association between the factors and the seniors’ interest in the three types of assistive apps. Smartphone-related dispositional traits were the strongest predictors across all three models. Among mobile phone usage patterns, smartphone use and the breadth of mobile phone features used were significant factors, while the significance of seniors’ personal characteristics and socio-economic conditions varied across the models. Hence, awareness that these factors play different roles in the acceptance of different assistive apps is needed in order to design viable interventions for their acceptance among seniors.

## 1. Introduction

Over the last few decades, there has been an increased interest in developing and deploying assistive technology (AT) that can support or enable seniors’ independent living. AT is an umbrella term for any device or system that allows an individual to perform a task they would otherwise be unable to do, or that which increases the ease and safety with which the task can be performed [1]. Early examples of AT include devices such as hearing aids, personal alarm buttons, and wheelchairs. Recently, smartphones have been identified as a promising platform for the deployment of AT [2]. Using smartphones for assistive purposes may offer several advantages [2,3]. First, smartphones offer flexibility in that users can install and remove various apps at will. Second, since they feature a built-in touchscreen that allows for the customization of the user interface to match users’ specific needs and skills, smartphones are potentially user-friendly [4]. Third, smartphones have various types of built-in sensors that can be employed for various purposes (e.g., accelerometers, global positioning system (GPS)). Finally, smartphones offer a range of connectivity options (e.g., Wi-Fi, near-field communication (NFC), mobile internet connection). These technological advantages make possible the development of applications for fall detection, healthy lifestyle promotion, communication with carers, medication adherence, and vital signs monitoring, among others [3,5,6,7,8]. In fact, it has been argued that assistive apps may represent a viable enhancement of traditional AT for independent living at home [2,8], and an increasing number of assistive apps are available in app stores [9].

While these developments are promising from a technological perspective, not much is known about seniors’ interest in using different types of assistive apps. In contrast with the abundant work on the acceptance of mobile health (m-health) services, such as apps that provide prevention, diagnosis, treatment, and/or monitoring of patient health status via mobile devices [10,11], and outcomes of m-health interventions, there is little evidence-based research on seniors’ acceptance of assistive apps. However, several lessons can be drawn from the existing literature on seniors’ acceptance of traditional AT and the literature on smartphone adoption by seniors. Reviews show that the frequency of use of traditional AT is related to personal characteristics (e.g., age, gender, diagnosis), the properties of the assistive device itself (e.g., quality, appearance, usability), the user’s environment (e.g., social support and physical barriers) and factors related to provisioning and implementation (e.g., training, installation process) [12,13]. However, these factors are related to the use of AT that seniors already own (i.e., post-implementation acceptance). The reasons why seniors start using technology can differ from their reasons for continuing the use of technology [14]. While studies drawing on technology acceptance models (TAM) have already acknowledged such limitations [15], TAM-based research on AT has rarely considered the multifaceted nature of seniors’ existing experience with AT and technology in general. For instance, the Senior Technology Acceptance and Adoption Model (STAM) does not include seniors’ prior experience with technology and their socio-demographic characteristics. Moreover, the role of facilitating conditions has been conceptualized to affect the actual use and not the behavioral intention of seniors’ use of technology [16]. In addition, a review of studies drawing on TAM or related models to explain technology acceptance among seniors found that specific biophysical limitations (e.g., cognitive and physical decline) and psychosocial conditions (e.g., social isolation and fear of illness) related to ageing were overlooked in prior research [17].

Taking into account these and similar concerns, the Cycle of Technology Acquirement by Independent-Living Seniors (C-TAILS) conceptual model [18] has recently been developed, based on longitudinal field research among independent-living seniors, which identifies six components of seniors’ specific needs and circumstances that may drive their motivations to start using a new technology for ageing in place. These include seniors’ current challenges related to independent living, their current use of technological and non-technological means, their internal technology-related schemas and attitudes, the external influences of the social network and organizations, and the physical environment in which seniors reside [18].

Previous research investigating the acceptance and use of smartphone devices and services among seniors points to various factors that pertain to several domains of the C-TAILS model. With reference to the challenges of independent living (e.g., maintenance of social resources, preservation of cognitive ability and mobility issues), it has been shown that smartphone use is to a great extent related to the personal characteristics of seniors (e.g., the instrumental activities of daily living (IADL), chronic diseases, age, and gender) and socio-economic conditions (e.g., education, household income, and living arrangements) [19,20,21]. In addition, the embracement of smartphone devices and services is associated with the intensity of seniors’ engagement with information and communication technology (ICT), requiring a variety of digital skills, including the intensity and breadth of mobile phone features used [5,19,22]. Relatedly, it has been indicated that smartphone-related beliefs and attitudes among seniors are conditioned by compatibility (i.e., the degree to which smartphones and AT are perceived as being consistent with existing values, past experiences, and the needs of potential adopters) [23], as well as negative emotions, such as mobile phone anxiety [10,19,24]. Another important aspect of the C-TAILS model is the resources (i.e., effort and money) that are required to start using new technology [18]. In the context of current research on smartphone acceptance, scholars refer to the latter as facilitating conditions, which involve not only encouraging services but also the normative obligations and expectations by the members of seniors’ social networks [21,24].

While past research has built an extensive and robust framework to understand the role of each group of factors in the adoption of smartphones and AT, currently there is inconclusive evidence on how these factors combine to predict seniors’ interest in assistive apps. Moreover, it is unclear whether these predictors can be generalized over a number of different assistive apps, or whether they demonstrate different predictive powers across different (groups of) assistive apps. We conducted a nationwide representative survey in Slovenia to fill these gaps in the current research, using explorative linear regression models to predict seniors’ interest in using three different types of assistive apps, based on four groups of factors derived from the C-TAILS model.

## 2. Materials and Methods 

### 2.1. Procedure

The research data were collected via a telephone survey during November and December of 2015. The target population was comprised of residents of Slovenia aged 55 and above. The decision to use 55 years of age as a threshold for inclusion in the study stems from the our understanding of ageing society, in which ageing refers not only to seniors as a specific population group, but also to the process of socially constructed meanings of age that accompany the changes in (social) roles assigned to people approaching a transition in their livelihood, marked by life-stage events such as retirement and withdrawal from the labor force. The initial sample of 6675 individuals in the target population with a landline or mobile phone number was obtained from the Central Population Register administrated by the Statistical Office of the Republic of Slovenia (SORS), using random sampling with stratification by age, gender, type of settlement (i.e., urban or rural), and region of residence. The number of individuals sampled within each stratum was proportional to the population size of the target population. The data collection resulted in 1581 completed survey interviews, which represented the total number of respondents (i.e., the realized sample). The final response rate (RR2) was 23.9% [25]. The data were weighted by age, gender, type of settlement and region using a raking method according to the population data provided by SORS to obtain a representative national sample structure. 

All respondents gave their informed consent for inclusion before they answered the survey questionnaire and participated in the study. According to the rules of the University of Ljubljana and the funding agency, the methods and subjects involved in this study were classified under research categories that are considered exempt from the Ethics Committee’s oversight.

### 2.2. Sample

The realized sample included 54.8% females, with 44.1% of respondents aged 55–64 years old and 6.6% of respondents aged 85 years or over (Table 1). In total, 14.9% of the respondents in the sample had some college or university education, 15.5% were still working, and 45.3% worked in high-skill occupations. The sample comprised 71.8% married respondents, while 19.8% were widowed, 20.2% said that they were living alone, and 50.8% lived in rural settlements with 2000 inhabitants or fewer. A total of 46.2% of the sample reported a net household income of 1100 € per month or less. In addition, 47.9% of the sample reported suffering from one or more chronic health conditions, while 8.3% were receiving help with (the instrumental) activities of daily living ((I)ADL).

### 2.3. Measures

#### 2.3.1. Dependent Variables

The questionnaire recorded the respondents’ interest in seven assistive smartphone apps that could potentially improve their quality of life domains in later life [8]. The exact wording of the question was as follows: ‘Given your current needs, how interested would you be in using [name of the selected assistive app] on a smartphone? Please express your interest on the scale from 1 to 5, where 1 means that you would not be interested in using it at all, and 5 means that you would be interested in using it very much’. If requested, a respondent was provided a brief definition and description of each assistive app (detailed descriptions of the assistive apps are shown in Table A1 in the Appendix A). During the analysis, the seven assistive apps were clustered into three groups, following Plaza et al.’s typologies [8] of social (i.e., video call), care (i.e., fall detection, SOS button, and GPS navigation) and health (i.e., in case of emergency (ICE), medication reminder, and monitoring physical activity). The respondents’ interest in the latter two groups of assistive apps was calculated by averaging the scores of the three assistive apps within each category.

#### 2.3.2. Independent Variables

Drawing on the C-TAILS conceptual model, the questionnaire included four groups of predictors related to the six components that cover seniors’ relationships with and motivations for using technology for independent living. In the first group, three items recorded seniors’ mobile phone usage patterns, namely what type of mobile phone the respondent used (i.e., feature phone or smartphone), whether they used a mobile phone daily or less often, and how many mobile phone features they used (e.g., texting, alarm clock, email). The second group contained a block of scale items assessing the smartphone-related dispositional traits of seniors. Compatibility with smartphones, smartphone anxiety, and facilitating conditions with reference to (potential) smartphone usage were adapted from prior studies [20,24,26]. These items were rated on a five-point scale from 1 (strongly disagree) to 5 (strongly agree). The three inventories showed acceptable reliability (compatibility, α = 0.85; smartphone anxiety, α = 0.60; facilitating conditions, α = 0.53) (for the exact wording of questionnaire items, see Table A2 in the Appendix A), with higher values indicating higher levels of compatibility, smartphone anxiety and facilitating conditions. The third group of predictors comprised four items in the questionnaire that measured the personal characteristics of seniors, including their gender, age, presence of chronic health conditions, and whether they needed help with (I)ADL. These variables were included following the C-TAILS model [18] and past studies [21,27], demonstrating their relevance for the acceptance of smartphone technology by seniors. Age and gender questions were adapted from Eurostat measures, the question about chronic health conditions was derived from Berglund et al. [28], and the items measuring (I)ADL were adapted from Katz [29]. The fourth group of predictors rated the socio-economic conditions of seniors. Items recorded the respondents’ educational attainment, occupation, household income and labor status, as well as whether the respondents were living at home alone and whether they were from rural or (semi-)urban areas. All the questions in this group were adapted from the respective Eurostat measures. In addition, education, household income and occupation were used to construct principal component analysis-based socio-economic status (SES) scores for the respondents, as explained by Petrovčič et al. [30].

### 2.4. Statistical Analyses

Descriptive statistical analyses were ran for sample demographic variables and univariate distributions of other dependent and independent variables in the regression models. In line with the research aim, three separate multiple linear regression models using the ordinary least squares (OLS) and enter methods were ran to assess the extent of which the independent variables predicted seniors’ interest in social-, care- and health-assistive apps. On the basis of the distribution of the responses of two multilevel categorical predictors (i.e., labor status and living area), these two variables were recoded into binary variables before they were used in the regression analyses. Since the dependent variables were administered only to smartphone users or feature phone users who had at least a little familiarity with smartphones (*N* = 752), and due to the respondents giving invalid (i.e., item non-response) answers on dependent and/or independent variables, the regression analyses were ran on 617 eligible respondents, who provided valid answers on all analyzed variables in the three models. The decision to delete units with item non-responses was informed by the non-significant (*p* > 0.1) results of Little’s test, which suggested that the missing values were missing completely at random. The IBM SPSS 22 software package (IBM, Armonk, NY, USA) was used to perform the data analyses.

## 3. Results

First, a descriptive outline of the distributions of the variables that were entered into the regression models is presented. Mobile phones were used by 89.8% of respondents in the realized sample, and 83.7% used them daily (Table 2). More than 81.3% respondents had heard about smartphones, with 61% among them having at least some familiarity with a smartphone. Among the mobile phone users, 27.0% used a smartphone, while 73.0% used a feature phone.

As Table 3 shows, the subsample of 617 respondents who were entered into the regression models reported using an average of five features on their mobile phones (M = 5.3, SD = 2.8) and expressed on average high agreement with the availability of facilitating conditions for (potential) smartphone use (M = 4.0, SD = 0.9), followed by a moderate compatibility of smartphone use with their lifestyle (M = 3.6, SD = 1.2) and a low level of smartphone anxiety (M = 1.9, SD = 1.0). In addition, on average, the respondents reported a high interest in the SOS button (M = 4.0, SD = 1.4), followed by ICE (M = 3.5, SD = 1.6), fall detection (M = 3.4, SD = 1.6), and GPS navigation (M = 3.3, SD = 1.7). The average level of interest in video calling (M = 2.6, SD = 1.6), physical activity monitoring (M = 2.6, SD = 1.6) and medication reminding (M = 2.6, SD = 1.6) was somewhat lower. This means that, on average, the respondents expressed (significantly, at the *p* < 0.001 level) highest interest in care-assistive apps (M = 3.6, SD = 1.1), followed by health- (M = 2.9, SD = 1.2) and social-assistive apps on smartphones (M = 2.6, SD = 1.6).

Since the C-TAILS conceptual model proposes that the six components which cover seniors’ relationships with and motivations for using technology for independent living are not hierarchically organized, the four groups of factors were entered into the regression model in one step. The first of the three multiple linear regression models revealed that the independent variables explained 13.8% of the variation in seniors’ interest in a social-assistive apps on smartphones (adjusted R^2^ = 0.138, F (616, 14) = 8.040, *p* < 0.01). On one hand, the number of features used on a mobile phone (β = 0.206, *p* < 0.01), compatibility (β = 0.193, *p* < 0.01), smartphone anxiety (β = 0.128, *p* < 0.01) and the respondent’s age (β = 0.101, 0.01 < *p* < 0.05) were positive significant predictors. On the other hand, facilitating conditions (β = 0.084, 0.05 < *p* < 0.1) and labor status (β = 0.084, 0.05 < *p* < 0.1) were marginally significant positive predictors (Table 4). Moreover, the independent variables explained 14.1% of the variance in seniors’ interest in care-assistive apps (adjusted R^2^ = 0.141, F (616, 14) = 8.225, *p* < 0.01). Daily use of a mobile phone (β = 0.090, 0.01 < *p* < 0.05), the number of features used on a mobile phone (β = 0.209, *p* < 0.01), compatibility (β = 0.237, *p* < 0.01), smartphone anxiety (β = 0.083, 0.01 < *p* < 0.05), and labor status (β = 0.114, 0.01 < *p* < 0.05) emerged as significant positive predictors in the model. Conversely, the negative values for the beta weights for smartphone use (β = −0.107, 0.01 < *p* < 0.05) and SES score (β = −0.099, 0.01 < *p* < 0.05) indicated that the respondents with smartphones and those with higher SES score were less likely to express interest in care-assistive apps when controlling for the values of all other predictors in the model. Finally, the results showed that the predictors under consideration were able to explain 8.9% of the variance in seniors’ interest in using health-assistive apps (adjusted R^2^ = 0.089, F (616, 14) = 5.291, *p* < 0.01). On one hand, the number of mobile phone features used by the respondents (β = 0.174, *p* < 0.01), smartphone anxiety (β = 0.090, 0.01 < *p* < 0.05) and the self-reported compatibility of seniors with smartphones (β = 0.228, *p* < 0.01), as well as the presence of at least one chronic health condition (β = 0.096, 0.01 < *p* < 0.05) were significant positive predictors. On the other hand, owning a smartphone (β = −0.136, *p* < 0.01), living alone (β = −0.080, 0.05 < *p* < 0.1), and the availability of better facilitating conditions to support smartphone adoption (β = −0.135, *p* < 0.01) significantly decreased the likelihood of interest in the adoption of health-assistive apps among seniors. 

## 4. Discussion

Looking at our results reporting seniors’ interest in various types of assistive apps, several observations can be made. On average, seniors expressed a very modest interest in assistive apps on smartphones, since the mean scores (on a 1–5 scale) were 3.6 for care-assistive apps, 2.9 for health-assistive apps and only 2.6 for social-assistive apps. These findings contrast with the favorable forecasts of many scholars, who see smartphones as a platform for an inclusive uptake of assistive services [2,31]. Our results did not support expectations that smartphones could quickly become gateways to health information or e-health interventions for seniors [32]. Conversely, our findings seem to corroborate observations of seniors being reluctant to adopt internet-based mobile services and apps [27,33], for example, empirical evidence shows that one in four individuals over 55 years of age has never downloaded a smartphone app [33]. Moreover, our results support previous evidence suggesting that ageing processes may lead seniors to consciously or subconsciously limit the number of apps they use [34]. This also supports Baltes’ concept of ‘selection’, which describes how seniors who are confronted with more options than their internal (i.e., cognitive ability) and external (i.e., support by others) resources can handle are forced to concentrate their energy on a subset of those options [35].

With reference to seniors’ experiences with technology, this study confirms the C-TAILS conceptual model, which differentiates between various aspects of mobile phone usage patterns and dispositional traits, such as technology-related attitudes and schemas. But even more importantly, our findings indicate that the role of one predictor may vary across different assistive apps. For example, while seniors who were using a larger number of features on their mobile phones reported a higher interest in all three types of assistive apps, the daily use of mobile phones increased seniors’ interest in only care-assistive apps. Hence, it is important to bear in mind that interventions focusing on the acceptance of different types of assistive apps among seniors might not be effective if each factor relating to seniors’ existing experience with mobile phone utilization is not carefully assessed and studied.

Quite unexpectedly, the results also showed that the usage of a smartphone does not necessarily facilitate an interest in assistive apps and that smartphone anxiety does not necessarily limit interest in assistive apps. Notably, even after controlling for other predictors in the model, smartphone users reported less interest in the adoption of care- and health-assistive apps, while seniors with higher smartphone anxiety showed significantly higher interest in the adoption of all three groups of assistive apps. Such findings invite us to speculate about possible explanations. Considering research investigating seniors’ acceptance factors of new smartphone features [22,36], a tentative suggestion could be that the existing smartphone users in our sample have had negative experiences with the design of smartphones. In particular, the touchscreen user interfaces of smartphones often do not meet the limited sensory, motoric and/or cognitive abilities of seniors, and they might become (more) reluctant to adopt any kind of smartphone-based services as a result [36]. Conversely, among seniors with higher smartphone anxiety, the importance of the unmet need that the assistive apps serve to fulfil might outweigh their potential concerns about smartphones. This explanation can be supported by previous literature demonstrating that seniors’ inexperience with and/or negative attitudes towards new assistive devices does not inhibit interest in their acceptance if the perceived care provision and assistance benefits are clear and understandable [37,38]. This seems to be the case, even more so for smartphone non-users, who expressed more interest in adopting assistive apps than smartphone users, who might have more concerns about their low self-efficacy in using such apps. In support of such reasoning, and the C-TAILS model, our study found that seniors with experience of a higher number of mobile phone features reported higher interest in all kinds of assistive apps. It seems that once the barriers related to problems with the usability of smartphones come down, and self-efficacy is established, seniors’ positive experiences of the usefulness and perceived value of more mobile phone features are likely to increase their interest in assistive apps.

Moreover, the importance of seniors’ smartphone-related dispositional traits is supported further by the significant effect of smartphone compatibility on expressed interest in all three kinds of assistive apps. In fact, in two out of the three regression models, smartphone compatibility was the strongest of all predictors. These findings are consistent with results in other studies, showing the direct positive effect of compatibility either on seniors’ (intention to) use smartphones [19,26] or on healthcare services and apps [23,39]. Thus, a viable strategy to increase seniors’ interest in assistive apps should be supported by approaches that develop their belief in the consistency of using smartphones in ways that embrace their existing values, needs, usage habits, and/or lifestyle. Conversely, the wider availability of facilitating conditions related to smartphone adoption and use (i.e., knowledge, social support, and economic resources) does not necessarily lead to higher interest in assistive apps. When controlling for all the other predictors in the models, facilitating conditions are only a marginally significant positive predictor of interest in social-assistive apps, and even a negative predictor of interest in health-assistive apps. With reduced costs and increased access to smartphones, having access to more social and economic resources for the use of smartphones does not necessarily translate into a higher interest in more advanced services on smartphones. Therefore, this finding also corroborates the C-TAILS model, which suggests that when seniors perceive the external influence of a social network as more of a normative pressure than a supportive resource, the ‘imposed’ or ‘externally driven’ motivational mechanisms might prevent their personal engagement with new assistive technologies for ageing in place [18]. 

Looking at personal characteristics related to the challenges of independent living, age, and the presence of chronic health conditions were each assessed as being a significant positive predictor of interest in social- and health-assistive apps, respectively. Additionally, gender and (I)ADL were not identified as significant predictors of any types of assistive apps. These findings support the C-TAILS model and resemble past research showing that, as they age, seniors become more interested in phone features that allow them to obtain direct and rich contact with providers of social support [40,41]. Moreover, seniors with a chronic health condition are more likely to consider a smartphone as a viable means of assistance. This finding is proven by the prior literature, confirming that health-related conditions should be considered among the most important predictors of m-health service uptake [27,32].

Predictors related to seniors’ socio-economic conditions might have unforeseen consequences for the acceptance of assistive apps. Namely, seniors living alone show negative attitudes toward health-assistive apps on smartphones. This may be because they perceive them as replacements and/or potential threats for the in-person provision of healthcare services. Similar conclusions were reached in studies on e-care services [42], where in-person contact with carers may be substituted by technology. This causes seniors to weigh the advantages of increased feelings of safety and relief against concerns about isolation. In particular, the interplay between the (perceived) advantages and disadvantages becomes important for seniors with assistive apps that enable carers to check and monitor the condition of care receivers remotely and/or when such technology limits their in-person contact with carers who provide them with multi-layered forms of assistance and support [41,42]. Next, socio-economic status (SES) has a negative impact on seniors’ interest in care apps. It could be suggested that more well-off seniors show less willingness to adopt care-assistive apps because they have enough resources to cover the expense of conventional care provision. Previous research suggested that independent-living seniors look at various competing technological and non-technological alternatives to meet their needs [18,42,43]. In this sense, well-off seniors might perceive a smartphone-based assistive app as a ‘sub-optimal’ solution for the fulfilment of their care needs because they have the necessary resources to pay for conventional forms of formal care and/or have access to more advanced ambient-assistive living technology. Conversely, seniors in low SES groups, who are in general more likely to receive formal and informal help [44], could perceive assistive apps as an (more) affordable solution to their care needs, with operational and economic advantages for themselves and their caregivers. With reference to seniors’ occupational status, our findings reinforce the suggestion that assistive apps support those who have to combine their job with informal care provision to significant others and, therefore, suffer from high levels of care burden [45]. Employed seniors may express an interest in assistive apps because they want to compensate their limited in-person availability with forms of mobile communication (e.g., video calls) and because they are aware of the potential risks associated with limited availability in situations that require immediate care assistance.

## 5. Limitations

These reported findings should be considered in the context of study limitations. First, the rather low response rate limits the generalizability of the results to the Slovenian population of seniors aged 55 or above. However, refusal reasons (i.e., time constraints or low relevance of the topic) did not differ from other studies, including those with a higher participation rate. Second, caution is advised in interpreting our results, since they may be related to the specific characteristics of seniors in Slovenia. While Slovenia is an average-performing European Union member state in terms of ICT benchmarks, seniors in Slovenia have some distinctive characteristics that might influence the relationships between the predictors investigated herein (e.g., a strong reliance on family for social support, bigger economic inequalities among seniors, and a higher percentage of retired people among seniors) [44]. Another country-specific characteristic relates to the low penetration of ICT-based AT, with only approximately 500 basic social alarm users in 2017 [42]. Such conditions might reverberate in a low awareness of the (benefits of) of assistive apps among seniors and their adult children in the role of informal carers. Limitations relate also to the selection of a limited set of assistive apps. We aimed to introduce the most typical assistive apps mentioned in the literature [8], as well as apps that could be most easily described to seniors with possibly no previous knowledge of the different types of assistive apps. Finally, this study explores only a limited set of predictors of interest in assistive apps drawn from the C-TAILS conceptual model. While we included four components (the challenges of independent living, current mobile phone use, internal smartphone-related schemas, and attitudes and physical environment in which seniors reside), we could also add the external influence of social network and organizations. This way the models might explain an even higher percentage of the variance of dependent variables. However, as with any exploratory regression model, a balance has to be found between being too stringent and too inclusive when deciding which predictors should be a subject of analysis. We attempted to include a broad range of acceptance predictors identified from different studies to sketch a baseline understanding of how different groups of predictors are related to the three types of assistive apps that have been suggested as having considerable potential for improving seniors’ quality of life while living independently at home.

## 6. Conclusions

An original feature of this study is that, based on the C-TAILS conceptual model, it compared the role of four groups of predictors of seniors’ interest in using different types of assistive apps. Our findings reveal that seniors’ modest interest in assistive apps is associated with a complex interplay of factors relating to their personal characteristics, technology-related dispositional traits, and social context. In addition, our results indicate that the role of these factors, when analyzed together, may be different for different types of assistive apps. To unveil the underlying mechanisms of interplay between these factors, further conceptual research is warranted in addition to dedicated intervention studies to facilitate the participation of seniors in the design of assistive apps by translating evidence-based research insights into design practice.

## Figures and Tables

**Table 1:** Sample characteristics (weighted data).

Variable	Categories	*N* ^a^	% ^c^
Gender	Male	1581	45.2
	Female		54.8
Age	55–64	1581	44.1
	65–74		29.6
	75–84		19.7
	85 or more		6.6
Education	Vocational or lower	1541	40.7
	High school		44.4
	College or university		14.9
Labor status	Active	1581	15.5
	Not active		84.5
Occupation	High skill	1459	45.3
	Low skill		54.7
Living area	Up to 500	1525	28.7
	501–2000		22.1
	2001–10,000		17.7
	10,001–50,000		13.9
	50,001 or more		17.6
Living alone	Yes	1532	20.2
	No		79.8
Marital status	Married or cohabiting	1537	71.8
	Single		8.5
	Widowed		19.8
Household income	Up to 700 €	1454	19.2
	701 to 1100 €		27.0
	1101 to 1500 €		22.1
	1501 to 2100 €		17.7
	2101 € +		14.1
Chronic health problem(s)	Yes	1552	47.9
	No		52.1
(I)ADL ^b^	Yes	1547	8.3
	No		91.7

Note: ^a^ Sample size varies due to non-responses and non-applicability of questions. ^b^ (I)ADL: (the instrumental) activities of daily living. ^c^ Percentages do not necessarily add up to 100 due to rounding.

**Table 2:** Mobile phone and smartphone usage sample characteristics.

Variable ^a^	Category	*N*	%
Mobile phone use (*N* = 1581)	Users	1420	89.8
	Non-users	161	10.2
Frequency of mobile phone use (*N* = 1405)	Daily or almost daily	1176	83.7
	Weekly or less often	226	16.3
Heard about smartphones (*N* = 1566)	Yes	1273	81.3
	No	293	18.7
Familiarity with smartphone (*N* = 1233)	Very low	481	39.0
	Low	237	19.2
	Neither low nor high	260	21.1
	High	187	15.2
	Very high	68	5.5
Mobile phone device (*N* = 1414)	Feature phone	1032	73.0
	Smartphone	382	27.0

Note: ^a^ Sample size varies due to non-responses and non-applicability of questions.

**Table 3:** Interest in assistive applications and agreement with smartphone-related dispositional traits.

Variables	Assistive Applications	M	SD	M ^a^	SD
Social-assistive application ^b^	Videocall	2.6	1.6	2.6	1.6
Care-assistive applications ^b^	SOS button	4.0	1.4	3.6	1.1
	Fall detector	3.4	1.6		
	GPS navigation	3.3	1.7		
Health-assistive applications ^b^	ICE	3.5	1.6	2.9	1.2
	Physical activity	2.6	1.6		
	Medication reminder	2.6	1.6		
Number of mobile phone features used		5.3	2.8		
Facilitating conditions ^c^		4.0	0.9		
Compatibility with smartphone ^c^		3.6	1.2		
Smartphone anxiety ^c^		1.9	1.0		

Note: N = 617. ^a^ The means are significantly different at the *p* < 0.001 level. ICE: In case of emergency. ^b^ Rating scale: 1, ‘not interested at all’ to 5, ‘very much interested’. ^c^ Rating scale: 1, ‘strongly disagree’ to 5, ‘strongly agree’.

**Table 4:** Multiple linear regression models predicting seniors’ interest in assistive applications.

Variables	Social-Assistive Applications			Care-Assistive Applications			Health-Assistive Applications		
	B	SE(B)	β	B	SE(B)	β	B	SE(B)	β
Mobile phone usage patterns									
Mobile phone device (1 = Smartphone)	0.100	0.156	0.031	−0.245	0.111	−0.107 **	−0.330	0.121	−0.136 ***
Daily mobile phone use (1 = Yes)	0.182	0.224	0.032	0.366	0.159	0.090 **	0.104	0.173	0.024
Number of mobile phone features used	0.119	0.029	0.206 ***	0.086	0.021	0.209 ***	0.075	0.023	0.174 ***
Smartphone-related dispositional traits									
Facilitating conditions	0.158	0.083	0.084 *	0.049	0.059	0.037	−0.192	0.064	−0.135 ***
Compatibility	0.262	0.063	0.193 ***	0.229	0.045	0.237 ***	0.232	0.049	0.228 ***
Smartphone anxiety	0.210	0.065	0.128 ***	0.097	0.046	0.083 **	0.111	0.050	0.090 **
Personal characteristics									
Gender (1 = Male)	0.025	0.127	0.008	0.003	0.090	0.001	−0.097	0.098	−0.04
Age	0.022	0.010	0.101 **	0.001	0.007	0.01	−0.011	0.008	−0.066
Chronic health problem(s) (1 = Yes)	−0.003	0.128	−0.001	0.147	0.091	0.063	0.238	0.099	0.096 **
(I)ADL (1 = Yes)	0.273	0.250	0.043	0.028	0.178	0.006	−0.136	0.193	−0.028
Socio-economic conditions									
Socio-economic status (SES) score	−0.114	0.078	−0.065	−0.124	0.055	−0.099 **	−0.049	0.060	−0.037
Labor status (1 = Active)	0.327	0.178	0.084 *	0.315	0.127	0.114 **	0.185	0.138	0.063
Living area (1 = (Semi)urban)	−0.009	0.130	−0.003	0.028	0.092	0.012	0.081	0.100	0.033
Living alone (1 = Yes)	−0.208	0.190	−0.045	−0.218	0.135	−0.066	−0.279	0.146	−0.080 *
Constant	−1.634	0.822		1.526	0.584		2.934	0.635	
Adjusted R^2^	−1.634	0.822	0.138	1.526	0.584	0.141	2.934	0.635	0.089
F (616, 14)	0.100	0.156	8.040 ***	−0.245	0.111	8.225 ***	−0.330	0.121	5.291 ***

Note: *N* = 617; the unstandardized beta (B), the standard error for the unstandardized beta (SE B), the standardized beta (β); *** *p* < 0.01 ** 0.01 < *p* < 0.05 * 0.05 < *p* < 0.1.

## Appendix A

**Table A1:** Description of assistive apps on smartphones.

Assistive App	Brief Description
Video call	Enables users to watch the interlocutors when talking with them on a smartphone.
SOS button	Allows users to call for help immediately (i.e., family member or emergency service) by pressing the button in case of need.
GPS navigation	Provides users with driving directions, localization of their geographical position on the map and/or pre-set points of interest.
Fall detector	Triggers an alarm when a fall is detected and sends a notification to family members, carers and/or professional staff.
In case of emergency (ICE)	Calls or sends out notifications to friends, family and/or professional staff containing all necessary personal information and users’ contacts in case of emergency.
Physical activity	Monitors users’ physical activity and records their calorie consumption, measures heart rate during physical activity and warns them in case of low activity rate.
Medication reminder	Allows users to enter data about all of their medicines. The name, photo, schedule and dose can be entered for each medicine. A reminder is triggered at a certain time for each medicine being entered in an app, which warns users that they need to take a drug.

**Table A2:** Questionnaire items for compatibility, smartphone anxiety and facilitating conditions.

Variable	Items for Smartphone Users	Items for Feature Phone Users
Compatibility	I believe that using a smartphone is suitable for me. I believe that using a smartphone fits my life style.	I believe that using a smartphone would be suitable for me. I believe that using a smartphone would fit my life style.
Smartphone anxiety	I feel apprehensive about using a smartphone. It scares me to think that I could break a smartphone.	I feel apprehensive about using a smartphone. It scares me to think that I could break a smartphone.
Facilitating conditions	I have enough money necessary to use a smartphone. I have the knowledge necessary to use a smartphone. A specific person (or group) can help me with smartphone difficulties.	I would have enough money necessary to use a smartphone. I would have the knowledge necessary to use a smartphone. A specific person (or group) could help me with smartphone difficulties.

Note: All items were measured on a five-point Likert-type scale, where 1 represented strong disagreement and 5 represented strong agreement.
